# Limits of Resolution and Sensitivity of Proton Detected MAS Solid-State NMR Experiments at 111 kHz in Deuterated and Protonated Proteins

**DOI:** 10.1038/s41598-017-07253-1

**Published:** 2017-08-07

**Authors:** Kai Xue, Riddhiman Sarkar, Carina Motz, Sam Asami, Diana C. Rodriguez Camargo, Venita Decker, Sebastian Wegner, Zdenek Tosner, Bernd Reif

**Affiliations:** 1 Helmholtz-Zentrum München (HMGU), Deutsches Forschungszentrum für Gesundheit und Umwelt, Ingolstädter Landstr. 1, 85764 Neuherberg, Germany; 2 Munich Center for Integrated Protein Science (CIPS-M) at Department Chemie, Technische Universität München (TUM), Lichtenbergstr. 4, 85747 Garching, Germany; 3 Bruker BioSpin, Silberstreifen 4, 76287 Rheinstetten, Germany; 4 Deptartment of chemistry, Faculty of Science, Charles University, Hlavova 8, 12842 Praha 2, Czech Republic

## Abstract

MAS solid-state NMR is capable of determining structures of protonated solid proteins using proton-detected experiments. These experiments are performed at MAS rotation frequency of around 110 kHz, employing 0.5 mg of material. Here, we compare ^1^H, ^13^C correlation spectra obtained from protonated and deuterated microcrystalline proteins at MAS rotation frequency of 111 kHz, and show that the spectral quality obtained from deuterated samples is superior to those acquired using protonated samples in terms of resolution and sensitivity. In comparison to protonated samples, spectra obtained from deuterated samples yield a gain in resolution on the order of 3 and 2 in the proton and carbon dimensions, respectively. Additionally, the spectrum from the deuterated sample yields approximately 2–3 times more sensitivity compared to the spectrum of a protonated sample. This gain could be further increased by a factor of 2 by making use of stereospecific precursors for biosynthesis. Although the overall resolution and sensitivity of ^1^H, ^13^C correlation spectra obtained using protonated solid samples with rotation frequencies on the order of 110 kHz is high, the spectral quality is still poor when compared to the deuterated samples. We believe that experiments involving large protein complexes in which sensitivity is limiting will benefit from the application of deuteration schemes.

## Introduction

Magic Angle Spinning (MAS) solid-state NMR for biological samples has taken a large leap forward during the past years^1–8^. Advancements in NMR technology such as the development of ultrahigh magnetic fields (ω_0_/2π > 1 GHz), emergence of ultrafast spinning probes (ω_r_/2π > 100 kHz), isotopic labelling strategies and development of new NMR methods are some of the key points behind this success. Solid-state NMR samples are spun in rotors at the magic angle (Arctan [√2] = 54.74°) with respect to the main magnetic field. The maximum achievable frequency of sample spinning at the magic angle is inversely proportional to the diameter of the solid-state NMR rotor^9^. Therefore, faster spinning rotors imply smaller volumes with less amount of sample and thus, a reduced sensitivity of the NMR spectra to start out with. Other parameters such as the diameter of the *rf* coil, detection of protons instead of heteronuclei may counteract the decrease of sample volume^10^.

Protons yield the highest sensitivity for detection due to their high gyromagnetic ratio. However, the strong proton-proton dipole couplings yield significant line broadening. Using deuterated samples helps to recover the spectral resolution while retaining high sensitivity. Several deuteration strategies have been proposed for solids during the last decade, that are now widely used. In general, deuteration strategies have to be adopted according to the available NMR hardware, in particular the achievable MAS frequency. Specific labelling schemes allow to study exchangeable as well as non-exchangeable protons. At a maximum MAS frequency of 24 kHz (3.2 mm MAS rotor, 10–20 mg sample), partial deuteration (H:D ≈ 20:80) at exchangeable amide sites in an extensively deuterated environment is required to obtain optimal spectral quality^11–13^. The emergence of 1.3 mm probes (2 mg of sample, maximum ~60 kHz MAS) allowed to employ 100% protons at exchangeable sites in an otherwise deuterated sample without having to sacrifice sensitivity or resolution^14–17^. On the other hand, the proton density in the aliphatic sidechain is significantly higher compared to the amide backbone. For such non-exchangeable protons, several fractional deuteration protocols have been implemented for solid-state NMR such as random deuteration at the aliphatic sites or RAP^18^, selective ILV-methyl labelling^19^, SAIL^20^, iFD^21^ and proton-cloud^22^ schemes. It was shown that fractional labelling of side chain protons yields excellent spectral resolution even at moderate MAS frequencies^23–27^. Finding the optimal deuteration scheme for non-exchangeable sites at a given MAS frequency poses a greater challenge in comparison to backbone amides, since deuteration itself limits the observation of side chain resonances. In addition, deuteration of a protein often significantly reduces protein expression yields^28^.

It has been suggested recently that sample rotation with a frequency of ~110 kHz (in 0.7 mm MAS rotor, 0.5 mg of material), which corresponds to the maximum MAS frequency achieved to-date, eliminates the need for deuteration to achieve high resolution^29, 30^. On the other hand, simulations indicate that MAS frequencies on the order of 250 kHz are necessary to yield comparable proton resolution^31^.

In this work, we compare ^1^H, ^13^C correlation spectra obtained for protonated and deuterated samples of a microcrystalline protein at a MAS rotation frequency of 111 kHz, and show that the spectral quality obtained from deuterated samples is superior to those acquired using protonated samples in terms of resolution and sensitivity.

## Results

**Figure 1:**
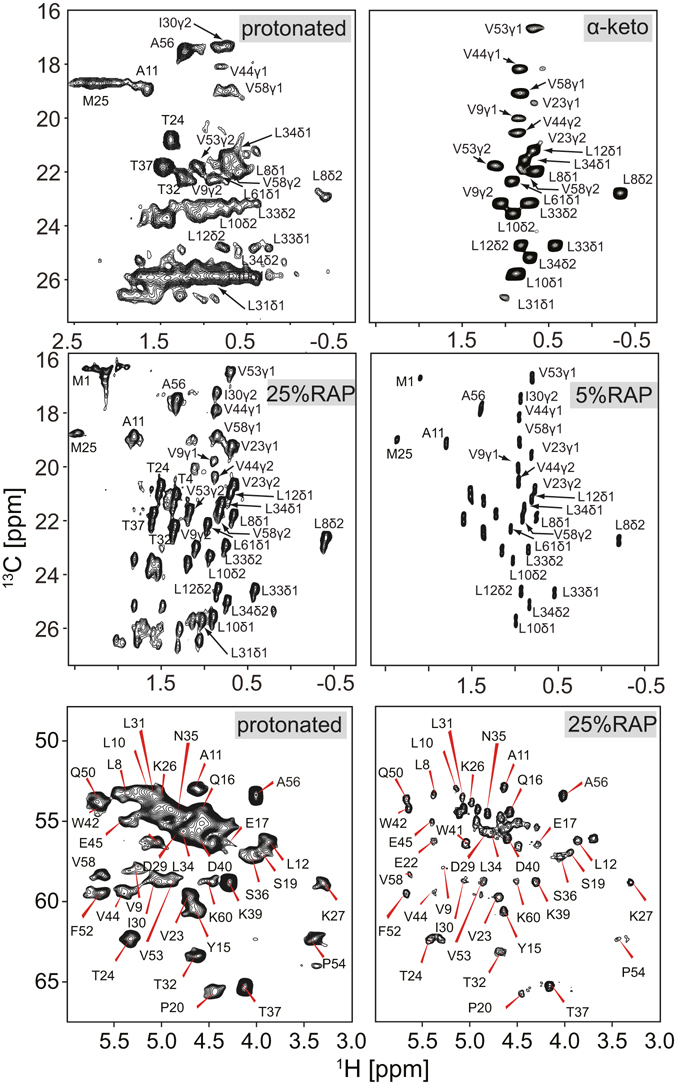
Comparison of MAS solid-state NMR ^1^H,^13^C correlation spectra obtained for protonated and deuterated samples of a microcrystalline α-spectrin SH3 domain. **Top**: Methyl region of the spectra from protonated (left) and α-ketoisovalerate (CH3) labelled (right) samples^33^ (recorded with B_0_ = 11.8 T at 111 kHz MAS frequency). **Middle**: Methyl region of ^1^H,^13^C correlation spectra from 25% (left, recorded with B_0_ = 20 T at 40 kHz MAS frequency) and 5% (right, recorded with B_0_ = 14.1 T at 20 kHz MAS frequency) RAP^24^ labelled samples. **Bottom**: Backbone H^α^,C^α^ correlation spectra for protonated (left) and 25% RAP (right) labelled samples.

Figure 1 shows the ^1^H,^13^C correlation spectra obtained for a protonated and two types of deuterated samples of a microcrystalline sample of the α-spectrin SH3 domain. The 25% and 5% deuterated RAP samples are prepared using u-[^2^H,^13^C]-glucose, and a D_2_O based M9 growth medium that has been diluted with 25% and 5% H_2_O to yield selective incorporation of protons at the aliphatic sites, respectively^24^. The SH3 sample in which pro-R or pro-S methyl groups of valine and leucine residues are specifically protonated was produced using α-ketoisovalerate as a precursor for amino acid biosynthesis, yielding CH_3_ isotopomers)^32, 33^.

We find that the methyl cross peak intensities in the ^1^H,^13^C correlation spectra obtained using the protonated sample vary significantly in comparison to the spectra obtained using the α-ketoisovalerate (CH3) labelled sample. A spectrum comprising the full spectral width is provided in the Supporting Information (Fig. S1). In particular, several methyl peaks are broadened or missing in the spectrum from the protonated sample. Using the RAP labelled samples, high resolution H^α^,C^α^ correlation spectra are obtained. The resolution of the spectrum from the 25% RAP labelled sample is only compromised due to the presence of three methyl isotopomers CH_3_, CH_2_D, CHD_2_ which yield isotope induced chemical shift changes^34^. This problem can be alleviated by using 5% RAP labelled sample. In the protonated sample, the α-carbon spectral region seems to be rather well dispersed at first sight. However, cross peaks significantly overlap in particular in the region of around 4.7/55 ppm proton/carbon chemical shifts.

**Figure 2:**
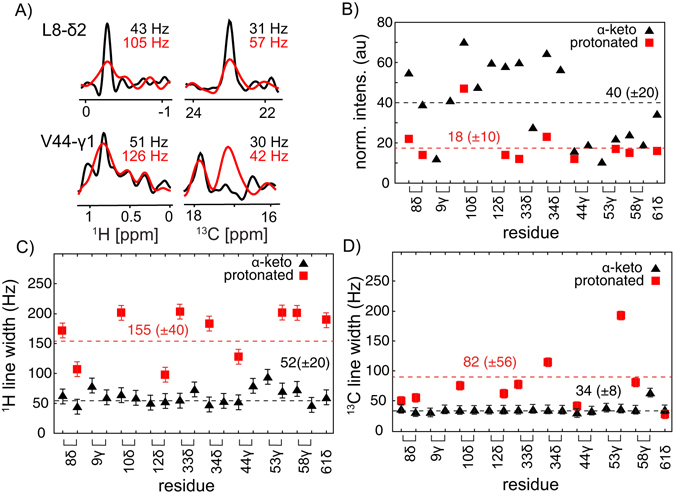
(**A**) 1D traces from the ^1^H,^13^C correlation spectra represented in Fig. 1 along the ^1^H and ^13^C dimension for residues L8 and V44. Spectra from the protonated and the α-ketoisovalerate labelled (CH3) sample are depicted in red and black, respectively. (**B**) Cross peak intensities for all rigid methyl residues in the protonated (red square) and deuterated (black triangle) α-spectrin SH3 domain. The error bar is within the symbol size. The intensities are normalised according to the sample amount as described in Fig S2. (**C**,**D**) ^1^H and ^13^C line width for all methyl bearing residues in the α-spectrin SH3 domain for the protonated (red square) and deuterated (black traingle) sample. The error of the line width is given by the acquisition times in the proton (35 ms) and the carbon (70 ms) evolution periods, respectively. The dashed lines represent the average line width for proton and carbon resonances, respectively. All experiments were carried out at B_0_ = 11.8 T (500 MHz ^1^H Larmor frequency).

To quantify the differences between the spectra, we analysed the experimental ^1^H and ^13^C line widths and the sensitivity in detail. The results are summarized in Fig. 2. Representative traces for two residues along the proton and carbon dimension of the correlation experiment are shown in Fig. 2A. Comparing the α-ketoisovalerate (CH3) labelled sample and the fully protonated sample, we observe an average gain in resolution on the order of 3 and 2 in the proton and carbon dimensions, respectively (Fig. 2C,D). To account for the difference in the amounts of material in the two rotors (Fig. S2), we quantified the amount of sample by performing 1D-^13^C experiments using direct ^13^C excitation. This ratio (~1.76 times) is used to normalize the cross peak intensities represented in Fig. 2B. In total, we find approximately a 2–3x higher sensitivity for the α-ketoisovalerate (CH3) labelled sample. This enhancement could potentially be further increased by an additional factor of 2 by making use of stereospecific precursors for amino acid biosynthesis^35^.

We attribute differences in peak intensities to the effective intra- and inter-methyl proton-proton dipolar couplings in the sample. To estimate the influence of the proton network around a given proton site, we quantified the spin density, following the convention of Zorin *et al*.^36^. In solids, a given spin is affected by many internuclear couplings of different magnitudes. Therefore, *d* ^RSS^, which is defined as the square root of the sum of squared dipolar couplings, provides a good approximation for an effective coupling experienced by a given spin

1 $${d}_{i}^{RSS}=\frac{{\mu }_{0}}{4\pi }{\gamma }_{H}^{2}\sqrt{\sum _{j}{(\frac{1}{{r}_{i,j}^{3}})}^{2}}$$

**Figure 3:**
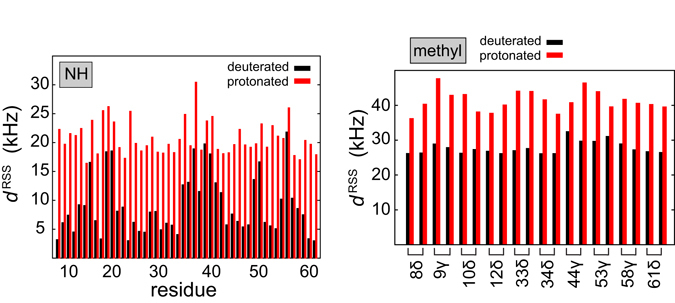
Effective ^1^H, ^1^H dipolar interactions *d* ^RSS^ for amide and methyl protons in the α-spectrin SH3 domain. The protonated and deuterated protein are represented in red and black, respectively. For the calculation of *d* ^RSS^ for amide protons in the deuterated sample, only exchangeable protons are assumed to be present, whereas for the protonated sample, both exchangeable and non-exchangeable protons were taken into account. *d* ^RSS^ has been calculated using a distance cut-off of 10 Å and (Eq. 1), employing the coordinate file 2nuz of the PDB database.

Figure 3 shows the *d* ^RSS^ values for amide and methyl protons in the α-spectrin SH3 domain (PDB-ID: 2nuz)^37^. In the calculation, a distance cut-off of 10 Å has been employed.

In the deuterated protein sample (back-substituted with 100% protons at exchangeable sites), the average proton, proton dipolar couplings for amides are on the order of 7 kHz, and thus approximately 3x smaller in comparison to the effective dipolar couplings in the protonated sample. In the deuterated protein sample, high *d* ^RSS^ values are obtained primarily in the turn regions (RT-loop around residue 20, the N-src-loop at residue 40, the distal loop with residues 47–49), for which short amide-amide distances are found. Apparently, a MAS rotation frequency of around 50 kHz is sufficient to yield efficient averaging of *d* ^RSS^ for the amide protons in perdeuterated samples. Under these conditions, average ^1^H^N^ line widths are on the order of 50 Hz^14^.

For the selectively methyl protonated sample, the effective proton, proton dipolar coupling is on the order of 27 kHz for methyl protons (Fig. 3). This value is similar in magnitude to the Hα *d* ^RSS^ values for a protonated sample (Figure S3). The ^1^H^α^,^13^C^α^ correlation spectrum for a protonated sample at a MAS rotation frequency of 111 kHz is reasonably well dispersed with an average proton line width on the order of 100 Hz (Figure S3), suggesting that this spinning frequency is sufficient to yield efficient averaging of *d* ^RSS^ in selectively methyl protonated samples. In contrast, the *d* ^RSS^ values for methyl protons in a fully protonated sample are approximately 1.5x larger, indicating that MAS frequencies on the order of 160 kHz and above are necessary to yield methyl ^1^H,^13^C correlation spectra with maximum resolution. Effective dipolar interactions are apparently not efficiently averaged in a protonated sample at a MAS frequency of 110 kHz. This in agreement with a previous study, where it has been suggested that homogeneous interactions require MAS frequencies that are significantly larger (10 times) than the strength of the interaction^31^.

**Figure 4:**
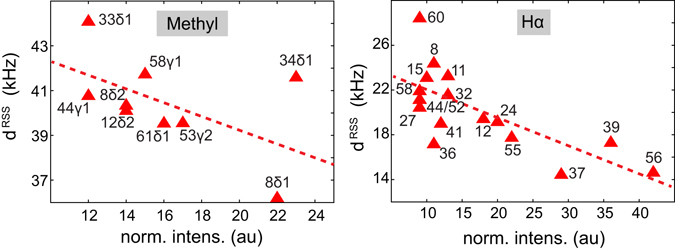
Correlation plot between the effective proton, proton dipolar coupling *d* ^RSS^ and the normalized peak intensities for methyl and Hα protons in a protonated SH3 sample. Experimental data were obtained from experiments that were recorded at a MAS rotation frequency of 111 kHz.

In order to test whether *d* ^RSS^ is a good descriptor for the proton spin network in MAS solid-state NMR, the calculated effective proton, proton dipolar couplings is represented as a function of the normalized cross peak intensities in Fig. 4. In general, higher Hα peak intensities are found for smaller *d* ^RSS^ values. Proton isotropic chemical shifts are not included in the analysis. *n* = *0* rotational resonance effects may potentially yield line broadening, which can be alleviated at higher magnetic fields. In a typical protein sample, the effective proton, proton dipolar interactions are on the order of 15–45 kHz. In that sense, rotation frequencies greater than 300 kHz are required in order to efficiently average all homonuclear proton dipolar couplings.

Even though fully protonated samples would be ideal for spectroscopy, a brute-force approach bears risks for structure determination protocols: Resonances of protons, which are involved in strong dipolar interactions, are potentially weak or not observable. These residues do not yield long-range distance restraints (>5 Å). If especially long-range distance restraints are missing, structure calculation protocols might potentially converge into a wrong fold.

## Conclusion

We demonstrated that at MAS rotation frequencies of 110 kHz, the spectral quality obtained from selectively methyl protonated samples is superior in comparison to the spectrum obtained from a fully protonated sample. The α-ketoisovalerate labelled (CH3) deuterated sample yields a gain in resolution on the order of 3 and 2 in the proton and carbon dimension, respectively. Sensitivity is enhanced by approximately a factor of 2–3, which could be further increased by a factor of 2 by making use of stereospecific precursors for amino acid biosynthesis. We believe that in particular experiments involving large non-symmetric protein complexes in which sensitivity is limiting will benefit from such deuteration schemes^38, 39^. The results presented here have been obtained only for a single microcrystalline protein preparation. In comparison to other samples, the α-spectrin SH3 domain yield very narrow line proton spectra. This allows to monitor subtle spectral changes induced by changes in the labelling scheme. We expect that similar effects will be observable for non-crystalline proteins as well, as the physical basis of dipolar interactions is the same, and the average proton density comparable for all proteins.

## Methods

The perdeuterated, selectively methyl group protonated microcrystalline SH3 sample was prepared as described previously^33^. In brief, expression was carried out in 100% D_2_O M9 medium supplemented with ^15^N-Ammonium Chloride and D-Glucose-^13^C, d_7_. α-ketoisovalerate (2-Keto-3-(methyl-d_3_)-butyric acid-4-^13^C, 3-d sodium salt, Sigma Aldrich) was added to the M9 minimal medium 1 h prior to induction with 1 mM IPTG (at OD_600_ = 0.5–0.6). yielding methyl CH3 isotopomers in a deuterated matrix. Subsequent to overnight expression, the SH3 domain was purified via anion exchange and size exclusion chromatography as described before. For crystallization, pure protein was lyophilized and dissolved in 100% D_2_O (final concentration: 8–10 mg/ml). Ammonium sulfate (dissolved in 100% D_2_O) was added to a final concentration of 100 mM and the pH was adjusted to 8.0 by adding NaOD. In order to produce the RAP labelled samples, the deuterated M9 minimal medium was supplemented with H_2_O (25% H_2_O and 5% H_2_O for 25% and 5% RAP samples, respectively)^24^. The protonated sample was prepared employing only protonated chemicals. The protein samples were not unfolded prior to crystallization, and no paramagnetic dopant was used in the crystallization buffer. The amount of protonation in the samples have been characterised in earlier work^18, 40^.

The proton detected CP based HSQC experiments for the protonated and α-ketoisovalerate labelled samples were carried out at a static magnetic field of 11.8 T (^1^H Larmor frequency 500 MHz), employing a 0.7 mm H/C/N MAS probe. The best Hartmann-Hahn condition was found at a nominal RF amplitudes of 70 kHz and 40 kHz on the ^1^H and ^13^C channel, respectively. A linear ramp on the ^1^H channel was applied for the ^1^H− > ^13^C CP step (contact time: 800 μs, range of amplitude: 70–100%) and for ^13^C− > ^1^H CP (contact time: 500 μs, range of amplitude: 100–70%), respectively. For hard pulses, B_1_ amplitudes of 156 kHz and 100 kHz were used for ^1^H and ^13^C, respectively. Low power decoupling was applied in the evolution periods using swept TPPM during t_1_ (ω_1H_/2π = 30 kHz)^41^, and WALTZ16 (ω_13C_/2π = 5 kHz) in the direct dimension. Water suppression was achieved using MISSISSIPPI with ω_1H_/2π = 30 kHz for 300 ms^42^. Phase-cycling was performed as described in Barbet-Massin *et al*.^17^. For all the experiments, the actual sample temperature was calibrated to 288 K using DSS (set temperature: 263 K, VT gas flow rate: 480 L/h). In all experiments, the acquisition times in the F1 and F2 dimensions were set to 70 ms and 35 ms (^13^C, ^1^H), respectively. The relaxation delays were set to 0.5 s and 1.5 s in the protonated and α-ketoisovalerate labelled sample, respectively.

The spectrum of the 25% RAP labelled sample was recorded at 20 T (850 MHz) setting the MAS frequency to 40 kHz. The spectrum of the 5% RAP sample was recorded at 14.1 T (600 MHz) with the MAS frequency adjusted to 20 kHz. For experiments involving RAP labelled samples, scalar coupling based HMQC type experiments were employed. 2–3 kHz of WALTZ16 decoupling was used during proton detection.

## Electronic supplementary material

Supplementary Information
