# Supplementary material for: Limits of Resolution and Sensitivity of Proton Detected MAS Solid-State NMR Experiments at 111 kHz in Deuterated and Protonated Proteins
Source: Sci Rep. 2017 Aug 7;7:7444. doi: 10.1038/s41598-017-07253-1 (PMC5547042; doi:10.1038/s41598-017-07253-1)
Supplement: Supplementary file 1 — Supplementary Information [file 41598_2017_7253_MOESM1_ESM.pdf]

Supporting Information for the manuscript

Limits of Resolution and Sensitivity of Proton Detected MAS Solid-State NMR Experiments at 111 kHz in Deuterated and Protonated Proteins

Kai Xue,[a] Riddhiman Sarkar,[a,b,*] Carina Motz,[b] Sam Asami,[b] Diana C. Rodriguez Camargo,[b] Venita Decker,[c] Sebastian Wegner,[c] Zdenek Tosner,[b,d] and Bernd Reif[a,b]*

March 20, 2017

[a] Helmholtz-Zentrum München (HMGU), Deutsches Forschungszentrum für Gesundheit und Umwelt, Ingolstädter Landstr. 1, 85764 Neuherberg, Germany

[b] Munich Center for Integrated Protein Science (CIPS-M) at Department Chemie, Technische Universität München (TUM), Lichtenbergstr. 4, 85747 Garching, Germany

[c] Bruker BioSpin, Silberstreifen 4, 76287 Rheinstetten, Germany

[d] Deptartment of chemistry, Faculty of Science, Charles University, Hlavova 8, 12842 Praha 2, Czech Republic

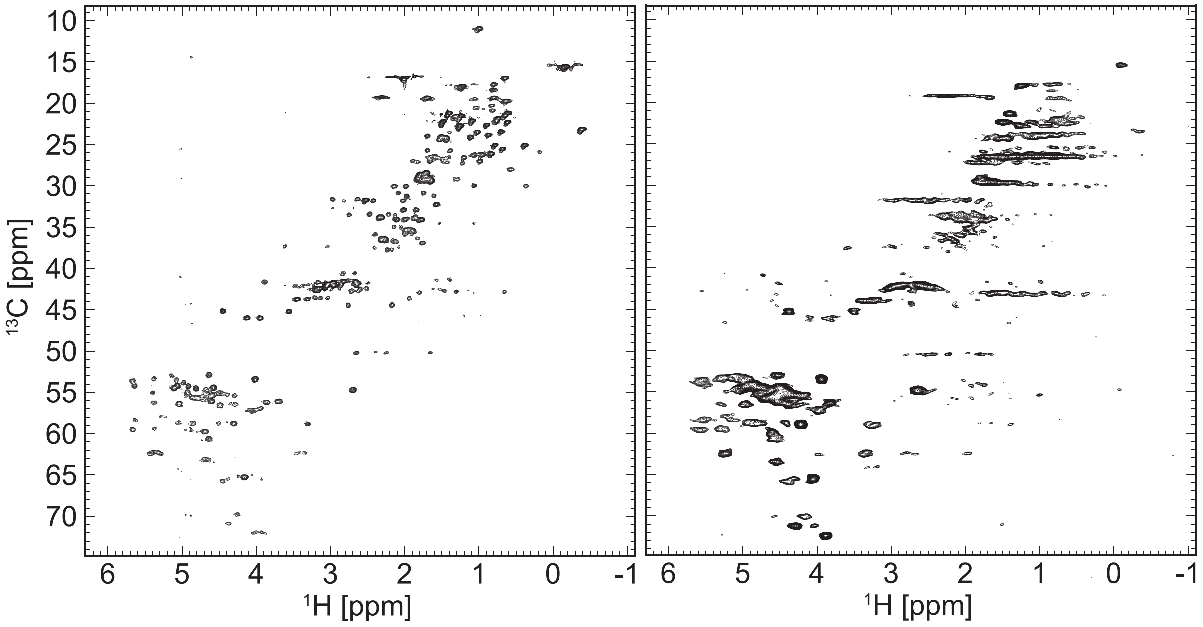

Figure S1. *Comparison of MAS solid-state NMR $^{1}H,^{13}C$ correlation spectra obtained for a fully protonated (right) and a 25 % RAP deuterated sample (left) of two microcrystalline SH3 domain preparations. The full aliphatic chemical shift range is shown. The spectrum for the fully protonated sample was recorded at 11.8 T (500 MHz), using a 0.7 mm MAS probe. The MAS rotation frequency was adjusted to 111 kHz. The 25 % RAP spectrum is represented for comparison. The spectrum of the 25% RAP labelled sample was recorded at 20 T (850 MHz), setting the MAS frequency to 40 kHz.*

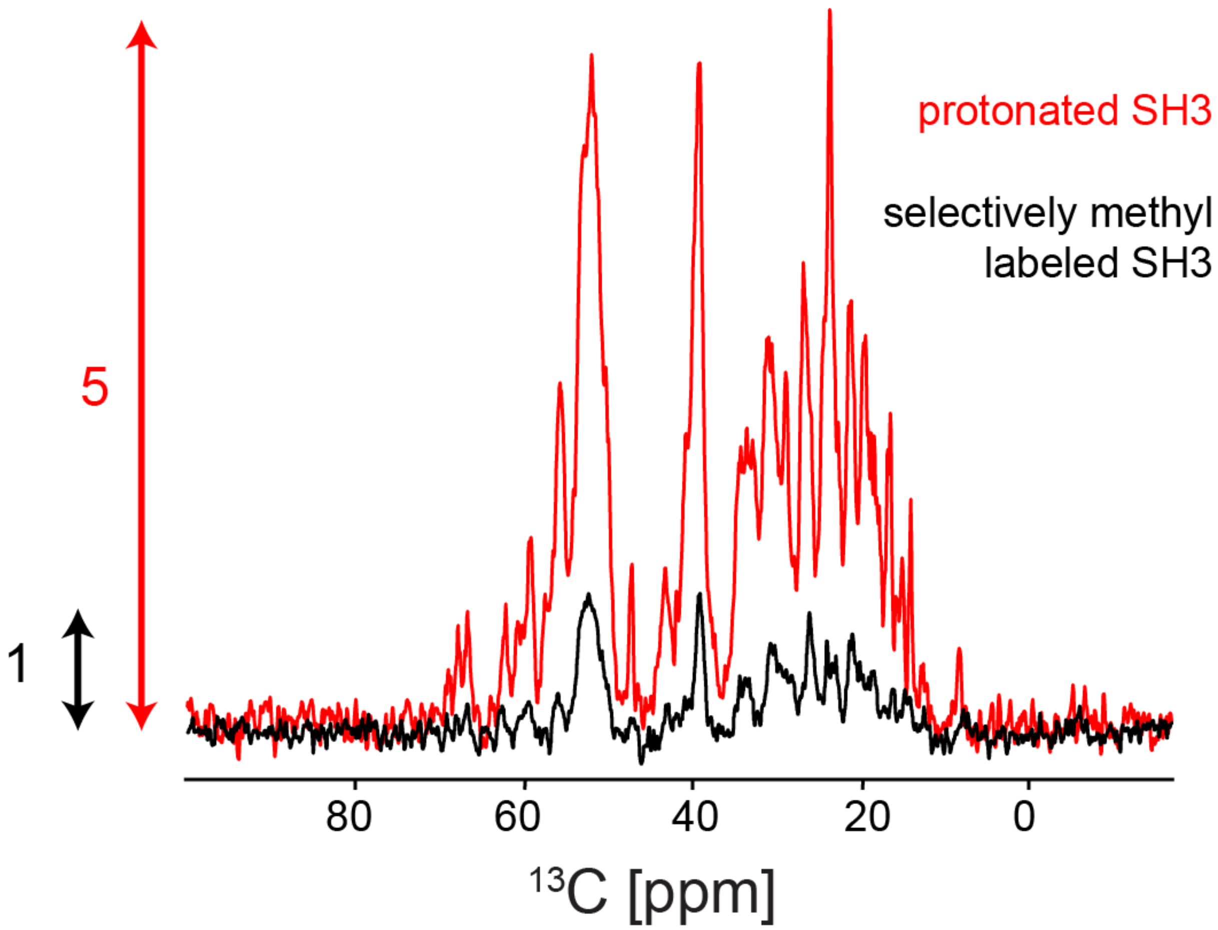

Figure S2. *Normalization procedure to determine the relative amount of material in the two 0.7 mm MAS rotors. One rotor contained protonated, microcrystalline SH3 protein, the second rotor was filled with deuterated, methyl protonated* (α-ketoisovalerate*) micro-crystalline SH3. For both samples, ^{13}C-1D experiments have been recorded using ^{13}C direct excitation (relaxation delay = 30s). The protonated sample was recorded using 512 scans, whereas the deuterated protein was recorded with only 64 scans. The signal intensity of the protonated sample is approximately 5 times larger in comparison to the intensity achieved in the deuterated sample. To yield a similar noise level, the spectrum of the deuterated sample was scaled with $\sqrt{8}$ to account for the different number of scans. In total, the protonated sample should thus contain 5 / $\sqrt{8}$ = 1.76 more material in comparison to the deuterated sample.*

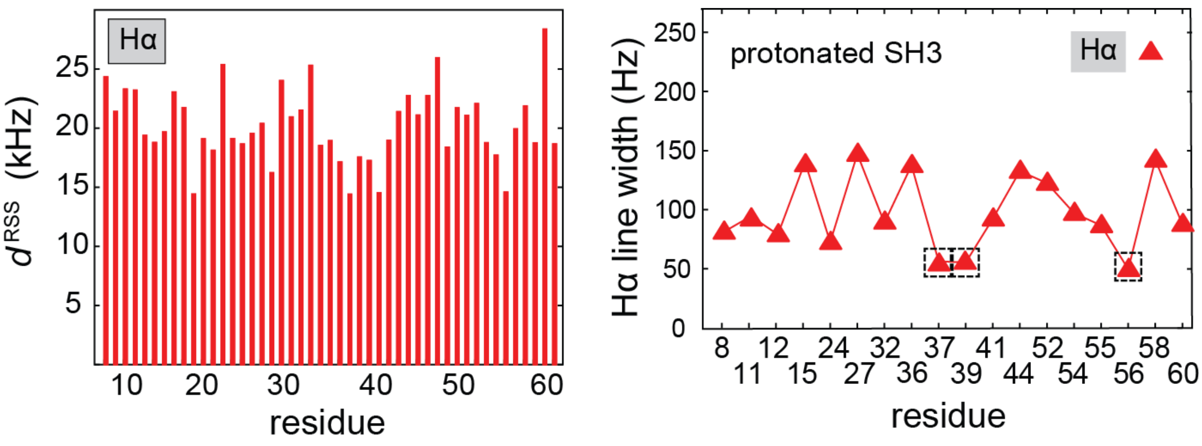

Figure S3. *Effective ^1H, ^1H dipolar interactions d^{RSS} for Hα protons in the α-spectrin SH3. d^{RSS} has been calculated using a distance cut-off of 10 Å and (Eq. 1) of the main manuscript. For the calculation, the coordinate file 2nuz of the PDB database has been employed.*
